# Fundc1 is necessary for proper body axis formation during embryogenesis in zebrafish

**DOI:** 10.1038/s41598-019-55415-0

**Published:** 2019-12-11

**Authors:** Gongyu Xu, Hao Shen, Emile Nibona, Kongyue Wu, Xiaomei Ke, Md. Abdullah Al Hafiz, Xiaoting Liang, Xueping Zhong, Qingchun Zhou, Chao Qi, Haobin Zhao

**Affiliations:** Hubei Key Laboratory of Genetic Regulation and Integrative Biology, School of Life Sciences, Central China Normal University, Wuhan, 430079 Hubei China

**Keywords:** Zebrafish, Body patterning, RNAi

## Abstract

FUN14 domain-containing protein 1 (FUNDC1) is a mitochondrial outer membrane protein which is responsible for hypoxia-induced mitophagy in mammalian cells. Knockdown of *fundc1* is known to cause severe defects in the body axis of a rare minnow. To understand the role of Fundc1 in embryogenesis, we used zebrafish in this study. We used bioimaging to locate zebrafish Fundc1 (DrFundc1) with MitoTracker, a marker of mitochondria, and/or CellLight Lysosomes-GFP, a label of lysosomes, in the transfected ovary cells of grass carp. The use of Western blotting detected DrFundc1 as a component of mitochondrial proteins with endogenous COX IV, LC3B, and FUNDC1 in transgenic human embryonic kidney 293 T cells. DrFundc1 induced LC3B activation. The ectopic expression of *Drfundc1* caused cell death and apoptosis as well as impairing cell proliferation in the 293 T cell line, as detected by Trypan blue, terminal deoxynucleotidyl transferase dUTP nick end labeling (TUNEL) and incorporation of BrdU. DrFundc1 up-regulated expression of both autophagy- and apoptosis-related genes, including *ATG5*, *ATG7*, *LC3B*, *BECLIN1*, and *BAX* in transgenic 293 T cells. A knockdown of *Drfundc1* using short hairpin RNA (shRNA) led to midline bifurcation with two notochords and two spinal cords in zebrafish embryos. Co-injection of *Drfundc1* mRNA repaired defects resulting from shRNA. Knockdown of *Drfundc1* resulted in up- or down-regulation of genes related to autophagy and apoptosis, as well as decreased expression of neural genes such as *cyclinD1*, *pax2a*, *opl*, and *neuroD1*. In summary, DrFundc1 is a mitochondrial protein which is involved in mitophagy and is critical for typical body axis development in zebrafish.

## Introduction

Mitochondria are essential for cellular energy metabolism and for controlling a series of key metabolic and biosynthetic pathways^1–3^. Fission and fusion of mitochondria regulate metabolism and embryonic development as well as autophagy and apoptosis of cells^4–6^. Mitochondrial dysfunction is the reason for many age-related diseases such as cancer^7^, deafness^8^, diabetes^9^, and neurodegenerative diseases (including Alzheimer’s and Parkinson’s diseases)^10^. Therefore, removal of dysfunctional or superfluous mitochondria by autophagy, known as mitophagy^11,12^, is crucial for survival.

During mitophagy, dysfunctional or superfluous mitochondria are incorporated into double-membrane structures known as autophagosomes, which are in turn delivered to lysosomes for degradation and reuse^13,14^. Interference with the expression of genes critical to mitophagy leads to severe defects. To give several examples, inactivation of *3-hydroxybutyrate dehydrogenase type 2* (*bdh2*) leads to mitochondrial dysfunction and mitophagy, which delays erythroid maturation in zebrafish^15^, cytosolic p53-mediated inhibition of mitophagy leads to heart failure in mice^16^, while in *Drosophila*, knockdown of *optic atrophy 1* (*opa1*) results in smaller mitochondria, developmental defects, and muscle degeneration^17^.

FUN14 domain-containing protein 1 (FUNDC1), a mitochondrial outer membrane protein in mammalian cells, plays a key role in mitophagy by binding to light chain 3 (LC3), an autophagy marker, via its LC3-interacting region (LIR) motif^18^. FUNDC1 overexpression induces mitophagy in several cell lines, including HeLa (cervical cancer cells), MCF-7 (breast cancer cells), and MEF (mouse embryonic fibroblasts)^19^. During mitochondrial fission, FUNDC1 is also an adaptor for dynamin-related protein 1 (DRP1). FUNDC1 overexpression induces mitochondrial fission, while knockdown of FUNDC1 results in mitochondrial fusion^18^. Under hypoxia, FUNDC1 accumulates at endoplasmic reticulum (ER)-mitochondria contact sites through interactions with Calnexin^20,21^. Up-regulation of FUNDC1 mediates proliferation and invasion of human ovarian cancer cells^22^. FUNDC1 also protects against cardiac ischemia-reperfusion injury (IRI) through mitophagy and interacts with receptor-interacting serine-threonine kinase 3 (Ripk3)^23,24^.

Authors have previously found that knockdown of *fundc1* causes severe defects in the body axis of a rare minnow (*Gobiocypris rarus*)^25^. However, the role of FUNDC1 in embryogenesis remains unclear. Therefore, zebrafish (*Danio rerio*) were used in this study due to their genomic sequences and well-documented embryonic development. We found that in zebrafish, Fundc1 (DrFundc1) is crucial for body axis formation.

## Results

### Location and function of DrFundc1 *in vitro*

Use of a multiple-sequence alignment of selected proteins as well as a molecular phylogenetic tree show that FUNDC1 is highly conserved in vertebrates (Fig. S1A,B). The open reading frame (ORF) of zebrafish *fundc1* (*Drfundc1*) is 459 base pairs (bp) in length. This encodes DrFundc1, a protein of 152 amino acids (AAs). DrFundc1 contains a putative LIR motif (YEVV) and a FUN domain (AA 51–132; Fig. S1A). DrFundc1 is a putative transmembrane protein with three α-helical stretches and the LIR motif in the N-terminal out of the mitochondrial outer membrane (Fig. S1C).

DrFundc1’s location and function *in vitro* were studied in two available cell lines: the grass carp (a relative of zebrafish and rare minnow in Cyprinidae family) ovary (GCO) cell line and the human embryonic kidney (HEK) 293 T cell line. This was due to a lack of both a proper zebrafish cell line for gene transfer and antibodies for detection of zebrafish proteins. We ask whether DrFundc1 is located in mitochondria and if it can work as its homolog FUNDC1 in mammalian cells to induce mitophagy.

Through the use of bioimaging, we found that the red fluorescence of DrFundc1-Cherry overlapped with the green fluorescence from MitoTracker Green (Thermo Fisher Scientific, Carlsbad, CA, USA; M7514), a reagent labeling mitochondrion, in transgenic GCO cells (Fig. S2A). Use of Western blotting showed a clear band (~44 kD) of DrFundc1-Cherry-His in the mitochondrial extract of transgenic GCO cells (Fig. S2B). It was also observed that DrFundc1-Cherry co-located with CellLight Lysosomes-GFP (Thermo Fisher Scientific; C10596), a reagent labeling lysosome, in transgenic GCO cells (Fig. S2C).

GCO cells grew poorly following *Drfundc1* transfection. The more *Drfundc1* was transfected, the poorer the cell growth (Fig. S2D). Cell numbers decreased significantly in the dosage of 400–500 ng of pCS2 + -Drfundc1-Cherry-His compared to control cells transfected with pCS2 + -Cherry plasmid. Cells transfected with *Drfundc1* displayed low density, while some cells were round in shape, floating, and aggregating into clusters.

**Figure 1:**
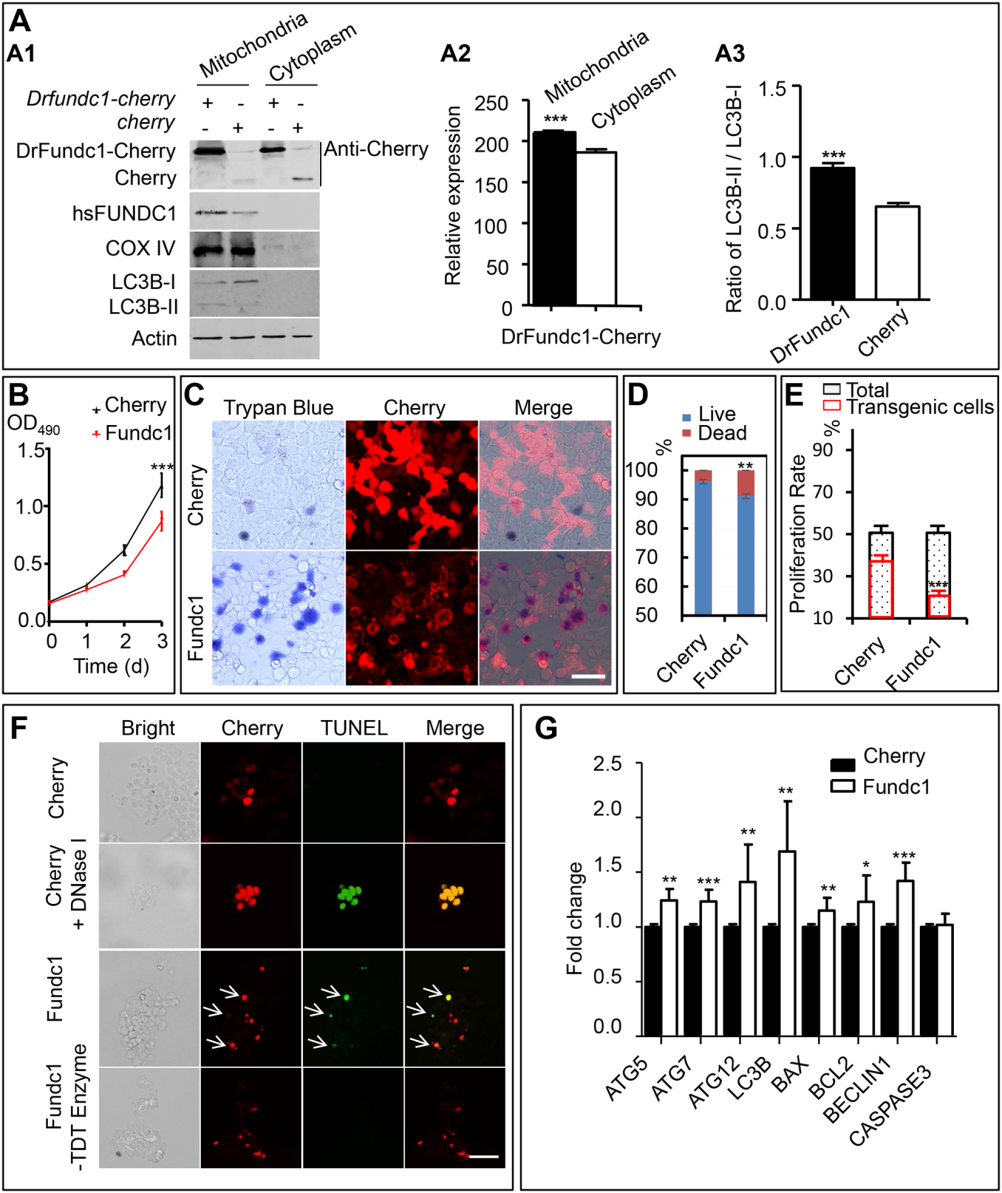
DrFundc1 reduced cell viability while inducing autophagy and apoptosis in transgenic 293 T cells. (**A**) Western blotting of proteins extracted from mitochondria and cytoplasm from transgenic 293 T cells transfected with pCS2 + -Drfundc1-Cherry-His as well as pCS2 + -Cherry plasmids. Antibodies used were anti-Cherry (detecting Drfundc1-Cherry-His and Cherry), anti-FUNDC1, anti-COX IV, anti-LC3B, and anti-β-ACTIN. (A1) Results of Western blotting. (A2) Gray-scale analyses of DrFundc1 using ImageJ in mitochondria and cytoplasm of cells transfected with pCS2 + -Drfundc1-Cherry-His. (A3) LC3B-II:LC3B-I ratio in mitochondria of transgenic cells transfected with pCS2 + -Drfundc1-Cherry-His and pCS2 + -Cherry. (**B**) DrFundc1 decreased viability of 293 T cells, measured by MTT assay. (**C-D**) DrFundc1 increased 293 T cell mortality, measured by Trypan Blue staining. (**E**) DrFundc1 decreased cell proliferation in transgenic cells, detected using BrdU incorporation. (**F**) DrFundc1 led to apoptosis of 293 T cells, detected using TUNEL assay. Arrows show apoptotic cells. Red indicates DrFundc1-Cherry or Cherry, while green indicates TUNEL-positive cells. Positive control: Cherry + DNase I. Negative control: incubation without TdT enzyme. (**G**) Expressional fold change of autophagy- and apoptosis-related genes, detected using qRT-PCR. *β-ACTIN* was used as an internal control. Significant differences between cells transfected with different plasmids are shown as asterisks. *P < 0.05; **P < 0.01; ***P < 0.001.

Use of Western blotting detected DrFundc1-Cherry fusion protein, mainly in the mitochondrial extract of transgenic 293 T cells which had been transfected with pCS2 + -Drfundc1-Cherry-His plasmid (Fig. 1A). DrFundc1-Cherry levels were significantly higher in mitochondria than in the cytoplasm of transgenic cells (*P* < 0.001), while endogenous cytochrome C oxidase subunit 4 (COX IV), LC3B, and endogenous FUNDC1 were primarily detected in mitochondria. Due to expression of DrFundc1 in the cells, more LC3B-I was converted to LC3B-II than in control cells (*P* < 0.001) (Fig. 1A). This suggests that DrFundc1 is a component of mitochondria and can lead to LC3 activation, which in turn causes mitophagy.

Deliberate expression of DrFundc1 was harmful to 293 T cells, which decreased in density, changed in morphology, aggregated, and even died following *Drfundc1* transfection (Fig. 1B,C, S3A,B). Use of a 3-(4,5-dimethylthiazol-2-yl)−2,5-diphenyltetrazolium bromide (MTT) assay found a significant decrease in proliferation of transgenic 293 T cells following *Drfundc1* transfection (*P* < 0.001) (Fig. 1B). Use of trypan blue staining and cell counting indicated a significant increase in mortality (*P* < 0.01) in cells by Drfundc1 expression (Fig. 1C,D). We used a 5-bromo-2′-deoxyuridine (BrdU) incorporation and found a decrease of cell proliferation in the transgenic cells with *Drfundc1* expression (*P* < 0.001), although no apparent changes to cell proliferation in total cells were observed (Fig. 1E, S3C). This suggests that DrFundc1 caused cell death and impaired cell proliferation.

Transfection of *Drfundc1* led to apoptosis of transgenic 293 T cells, as revealed by terminal deoxynucleotidyl transferase dUTP nick end labeling (TUNEL) assay. TUNEL-positive cells were observed in cells transfected with *Drfundc1*-*Cherry* (Fig. 1F), while use of a quantitative reverse transcription polymerase chain reaction (qRT-PCR) demonstrated expressional change of autophagy- and apoptosis-related genes in cells. Autophagy-related genes *ATG5*, *ATG7*, *ATG12*, and *LC3B*, as well as apoptosis-related genes *BCL2-associated X* (*BAX*), *B-cell CLL*/*lymphoma* 2 (*BCL2*), and *BECLIN1* were significantly up-regulated by use of DrFundc1 (Fig. 1G). Therefore, decreased cell viability was due to DrFundc1-induced autophagy and apoptosis. However, *CASPASE3* expression did not change, suggesting that apoptosis may not depend on *CASPASE3*.

### Expression of *Drfudnc1* in adult tissues and embryos of zebrafish

Use of a qRT-PCR detected *Drfundc1* in selected tissues (brain, eye, heart, intestine, liver, muscle, kidney, testis, and ovary; see Fig. S4A). Expression of *Drfundc1* was highest in the brain, followed by a moderate expression in the liver, ovary, testis, and kidney, while the lowest expression was in the heart and muscle. Use of a qRT-PCR also detected *Drfundc1* in zygotes throughout zebrafish embryogenesis (Fig. S4B). Expression of *Drfundc1* increased from the 1-cell stage, peaked at the gastrula stage (6 h post fertilization [hpf]), decreased at 12 hpf, and was then maintained at a low level from 24 hpf until hatching. We further studied the expression pattern of *Drfundc1* using a whole-mount *in situ* hybridization (WISH; see Fig. S4C). Use of WISH detected *Drfundc1* in embryos from zygote until hatching. *Drfundc1* was found in all blastomeres at early stages, from the 1-cell stage to the gastrula stage. Expression of *Drfundc1* was enriched in embryos’ heads – including brains and eyes – from 24 hpf onwards.

### Knockdown of *Drfundc1* caused serious defects in the body axis

**Figure 2:**
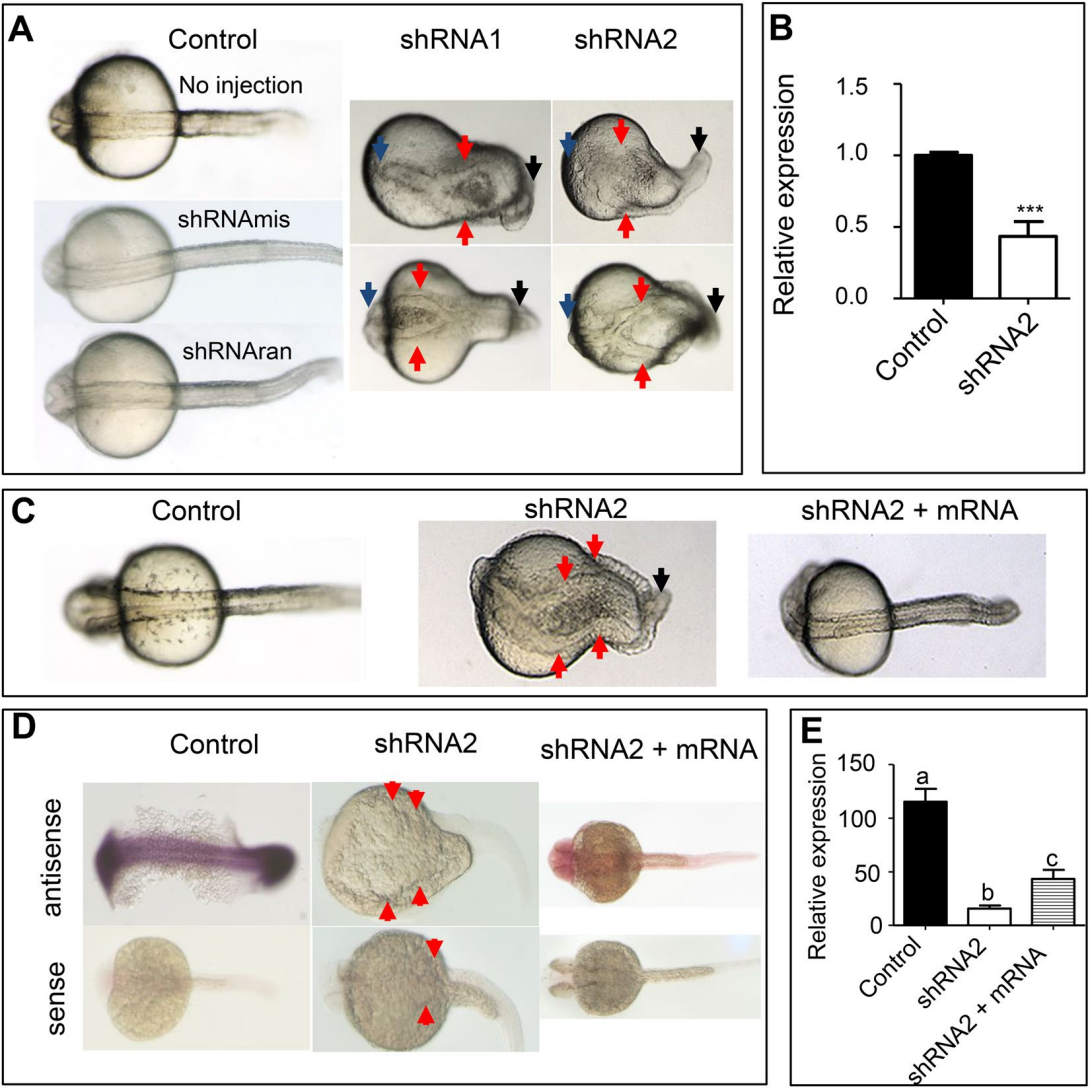
Knockdown of *Drfundc1* caused abnormal body axis development in zebrafish embryos (**A**,**B**), which was repaired by *Drfundc1* mRNA (**C**–**E**). (**A**) Midline bifurcation and headless embryos caused by microinjection of *Drfudnc1* shRNA1 or shRNA2 into zygotes at 24–28 hpf. (**B**) shRNA2 significantly decreased endogenous *Drfundc1* in the embryos (***P < 0.001), as measured by qRT-PCR with normalization by *β-actin*. (**C**) Co-injection of *Drfundc1* mRNA repaired defects caused by shRNA2. Before injection, 200 pg/nL of shRNA2 was mixed with 400 pg/nL of mRNA. Control embryos were not injected. (**D**) WISH results of *Drfundc1* in embryos with different treatments. (**E**) Gray-scale analysis of WISH results of *Drfundc1* (**D**), measured using ImageJ. Letters a, b, and c indicate significance (*P* < 0.05). All embryos were oriented with a dorsal view. Arrows point to abnormal sites in the embryos.

Knockdown of *Drfundc1* was induced by microinjecting specific *Drfundc1* short hairpin RNAs (shRNAs: shRNA1 and shRNA2) into zygotes. Both *Drfundc1* shRNA1 and *Drfundc1* shRNA2 caused similar defects in the body axis; that is, bifurcation in the midline, short body length, curved tail, and/or head dysplasia (Table 1 and Fig. 2A). Abnormalities were significantly high in embryos which had been microinjected with *Drfundc1* shRNAs (10.15–17.67%) compared to embryos with/without microinjection of the pSuper-puro plasmid and control shRNAs (mismatched shRNA [shRNAmis] or random shRNA [shRNAran]) (1.75–3.77%) (*P* < 0.05). *Drfundc1* shRNA2 caused higher abnormality rates in embryos than shRNA1. We therefore used *Drfundc1* shRNA2 for subsequent experiments.

**Table 1:** Rates of abnormal embryos.

Experiment	Group	Total embryos	Abnormal embryos	Abnormal rate % (mean ± SE)
1	No injection	3146	55	1.75 ± 0.08 ^a^
	pSuperpuro	2742	103	3.75 ± 0.11 ^a^
	shRNAmis	1326	50	3.77 ± 0.35 ^a^
	shRNAran	1146	20	1.75 ± 0.02 ^a^
	shRNA1	3566	362	10.15 ± 0.30 ^b^
	shRNA2	7153	1264	17.67 ± 1.52 ^c^
2	Control	2888	55	1.90 ± 0.09 ^a^
	shRNA2	3098	505	16.30 ± 0.58 ^b^
	shRNA2 + mRNA	2205	104	4.72 ± 0.31 ^c^

Note: Significant differences between groups are shown by different letters following the data (*P* < 0.05). The shRNAs – shRNA1, shRNA2 – target nucleotide 77–99 and 217–237 of *Drfundc1* ORF respectively. shRNAmis, mismatched shRNA; shRNAran, random shRNA without any target gene.

Compared to control embryos, endogenous *Drfundc1* decreased to 43.28% in embryos which had been microinjected with shRNA2 (Fig. 2B). To examine whether *Drfundc1* messenger RNA (mRNA) could repair *Drfundc1* shRNA-induced abnormalities, *Drfundc1* mRNA was co-injected with *Drfundc1* shRNA2 into zygotes, which developed typically (Fig. 2C). *Drfundc1* expression was highest in control embryos, followed by a moderate expression in embryos co-injected with *Drfundc1* mRNA and shRNA2, while only traces were detected in those microinjected with *Drfundc1* shRNA2 (Fig. 2D,E). Co-injection of *Drfundc1* mRNA and shRNA2 decreased abnormality from 16.30% (by *Drfundc1* shRNA2 microinjection) to 4.72% (*P* < 0.05) (Table 1). This suggests that *Drfundc1* mRNA repairs defects caused by *Drfundc1* shRNA2.

### Knockdown of *Drfundc1* formed two notochords and two spinal cords as well as affecting expression of autophagy-, apoptosis-, and neural development-related genes

**Figure 3:**
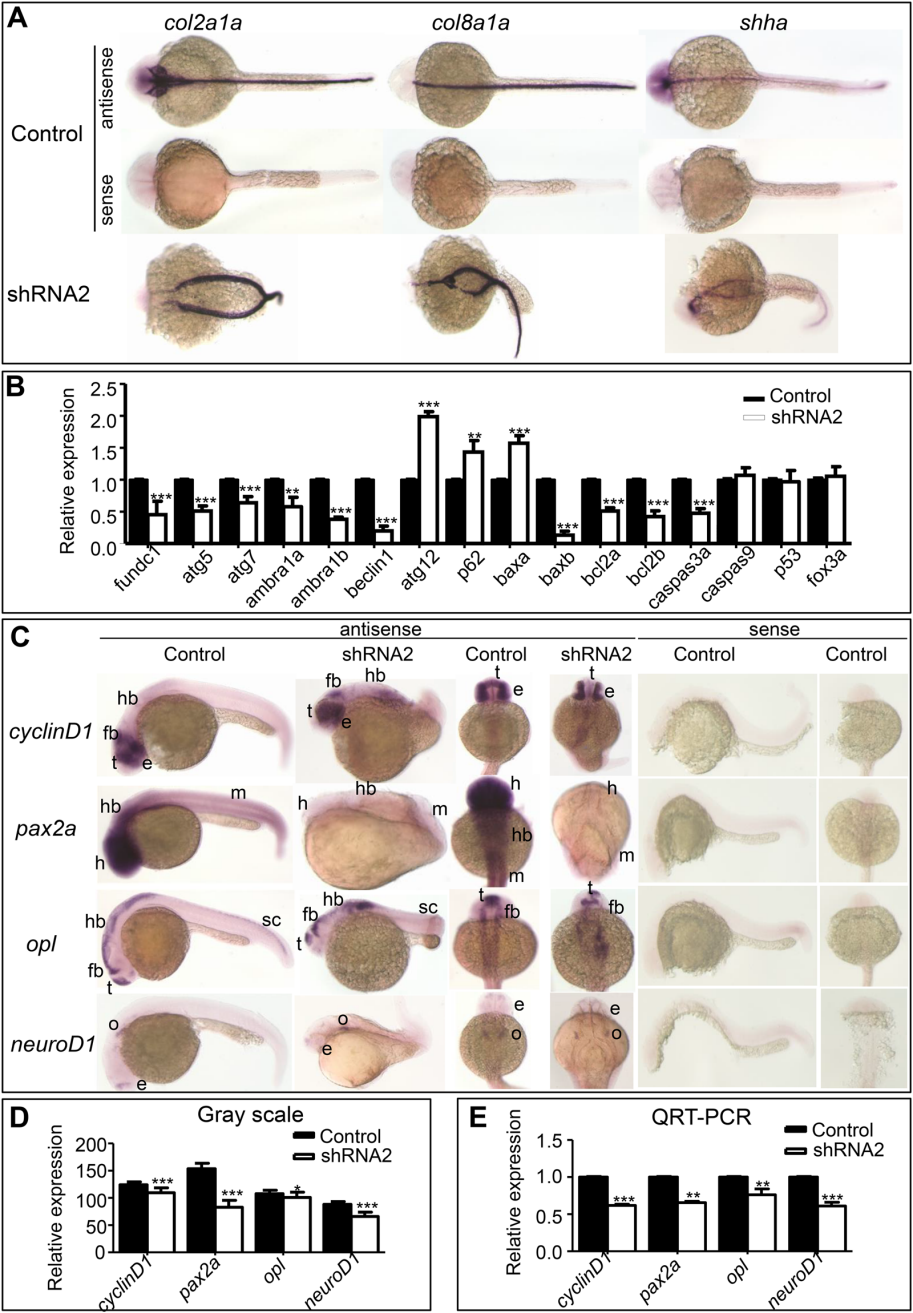
Expression of genes related to body axis formation, autophagy, and apoptosis. (**A**) WISH of *col2a1a*, *col8a1a*, and *shha* in embryos at 24–28 hpf. (**B**) Expression of autophagy- and apoptosis-related genes in embryos at 24–28 hpf, detected using qRT-PCR. *β-actin* was used as an internal control. (**C**) WISH of *cyclinD1*, *pax2a*, *opl*, and *neuroD1*, which are involved in neural system development. (**D**) Gray-scale analysis of WISH results of genes in (**C**), whose signal strength was measured using ImageJ. (**E**) Expression of *cyclinD1*, *pax2a*, *opl*, and *neuroD1* in embryos, detected using qRT-PCR, was normalized using *β-actin*.

To understand midline bifurcation, we subjectively studied *collagen 2a1a* (*col2a1a*) and *col8a1a* as notochord markers and *sonic hedgehog protein a* (*shha*) as a spinal cord marker^26–29^. Use of WISH clearly demonstrated that interference with *Drfundc1* induced formation of two notochords as well as two spinal cords in the embryo, while the control embryo was typical, with one notochord and one spinal cord (Fig. 3A), suggesting that midline bifurcation is a malformation of the body axis.

We hypothesized that insufficient mitophagy may be the reason for midline bifurcation due to *Drfundc1* knockdown. Authors of several studies have claimed that autophagy tends to be accompanied by a certain degree of apoptosis^30,31^. We therefore measured expressions of the following autophagy- and apoptosis-related genes: *atg5*, *atg7*, *atg12*, *autophagy and beclin 1 regulator 1a* (*ambra1a*), *ambra1b*, *nucleoporin 62* (*p62*), *beclin1*, *bcl2a*, *bcl2b*, *baxa*, *baxb*, *caspase3a*, *caspase9,* *p53*, and *forkhead transcription factor 3a* (*fox3a*)^32,33^.

Use of a qRT-PCR found that *atg5*, *atg7*, *ambra1a*, and *ambra1b* were down-regulated, while *p62* and *atg12* were significantly up-regulated by interference of *Drfundc1* (*P* < 0.01 or 0.001; Fig. 3B). In addition, *beclin1*, *bcl2a*, *bcl2b*, and *caspase3a* were significantly down-regulated (*P* < 0.001) by interference of *fundc1*. Interference of *fundc1* down-regulated *baxb* but up-regulated *baxa* (*P* < 0.001) and did not affect expression of *caspase9*, *p53*, and *fox3a*. This suggests that knockdown of *Drfundc1* interfered with autophagy and apoptosis, leading to body axis malformation in zebrafish embryos, and that this process depends on Caspase3 rather than Caspase9.

Neural system-related genes *cyclinD1*, *paired box 2a* (*pax2a*), *odd-paired-like* (*opl*), and *neuroD1* were detected using WISH and qRT-PCR. C*yclinD1* is associated with cell proliferation in the spinal cord^34^, *pax2a* is a mesencephalic marker participating in midbrain-hindbrain boundary development^35,36^, *opl* is a marker for forebrain cell fate which controls midline formation and forebrain patterning^37,38^, while *neuroD1* is critical for neuronal cell fate^39^. Use of WISH indicated that *Drfundc1* knockdown attenuated expression of *cyclinD1* in the telencephalon, *opl* in the spinal cord, *pax2a* in the head and muscle, and *neuroD1* in the eye (Fig. 3C). We used gray-scale analyses to find that gene expression significantly decreased in embryos which had been microinjected with *Drfundc1* shRNA2 (Fig. 3D). Subsequently, qRT-PCR confirmed *cyclinD1*, *pax2a*, *opl*, and *neuroD1* down-regulation due to *Drfundc1* knockdown (Fig. 3E).

## Discussion

We identified DrFundc1 as a mitochondrial protein associated with mitophagy. Additionally, DrFundc1 is critical for proper body axis formation in zebrafish. Knockdown of *Drfundc1* causes down- or up-regulation of autophagy-, apoptosis-, as well as neural development-related genes.

DrFundc1 was co-localized with MitoTracker, LC3B, COX IV, and FUNDC1 in transgenic GCO and 293 T cells. Deliberate expression of DrFundc1 caused conversion of LC3B-I to LC3B-II, which induced autophagy^40^. Co-localization of DrFundc1 with CellLight Lysosomes-GFP indicated occurrence of mitophagy. This suggests that DrFundc1 is a mitochondrial protein which is responsible for mitophagy.

In this study, ectopic DrFundc1 induced cell death through both autophagy and apoptosis. Use of a TUNEL assay demonstrated that apoptosis occurred in cells transfected with *Drfundc1*. Expression of autophagy- and apoptosis-related genes, including *ATG5*, *ATG7*, *ATG12*, *LC3B*, *BECLIN1*, *BAX*, and *BCL2* increased due to *Drfundc1* expression. As mammalian FUNDC1, DrFundc1 may regulate mitophagy via interaction with LC3^18–20,40–43^. Conversion of LC3-I to LC3-II by Fundc1 activates the autophagy pathway. Following this, ATG7 increases and activates ATG12, which binds to ATG5 and BECLIN1, forming an autophagy complex^42,43^. Finally, autophagy occurs, which in turn induces apoptosis via BAX, a pro-apoptotic factor, and BCL2, a survival factor^44,45^. Cell death can result from apoptosis and/or autophagy.

Mitophagy is pivotal for the survival of organisms while dysfunction of mitochondria causes severe deficiencies in zebrafish^15^, mice^16^, and fruit flies^17^. Zebrafish Fundc1 is involved in mitophagy, as described above. In this study, *Drfundc1* knockdown caused severe defects in the body axis, such as midline bifurcation and headlessness, which had been previously observed in rare minnows^25^. Co-injection of *Drfundc1* mRNA repaired these defects, although *Drfundc1*’s expression level did not go back to typical levels. This suggests that Fundc1 is necessary and critical for typical embryogenesis in fish.

The genes *atg5*, *atg7*, *ambra1a*, *ambra1b*, *beclin1*, *p62*, and *atg12* code major proteins for autophagy^30,31,40–46^. In mammals, down-regulation of *ATG5* family genes as well as up-regulation of *P62* lead to insufficient autophagy^42,43,46^. ATG12 is crucial for autophagosome elongation, but requires activation by ATG7. ATG12 conjugates with ATG5, forming a complex^42,43^. In this study, *p62* was increased, while *atg5*, *atg7*, *ambra1a*, *ambra1b*, and *beclin1* expression was decreased in zebrafish embryos due to interference with *Drfundc1*. This indicates that knockdown of *Drfundc1* resulted in insufficient autophagy in zebrafish embryos, although *atg12* expression was increased.

Apoptosis is associated with mitophagy^30,31^. Knockdown of *Drfundc1* decreased expression of the apoptosis-related genes *baxb*, *bcl2a*, *bcl2b*, and *caspase3a*^32,33,45,47,48^, contrasting to results of 293 T cells due to ectopic expression of *Drfundc1*. This means that *Drfundc1* knockdown interferes with apoptosis in zebrafish embryos. Apoptosis is a major part of typical development in many organisms, including zebrafish^49^. Interference with the process of apoptosis is likely to result in defects.

Midline bifurcation had been reported in cases of forced expression of insulin-like growth factor 2a (IGF-2a)^27^ as well as loss of Squint^28^ in zebrafish. Overexpression of IGF-2a induced Akt phosphorylation and caused midline bifurcation^27^. Loss of Squint, a nodal-related protein, resulted in midline bifurcation due to the failing expression of *wnt5b*^28^. Nodal regulatory factors contribute to mitochondrial homeostasis^50^. Nodal factors can down-regulate phospho-Akt while activating Smad2 and mitophagy to induce granulosa cell apoptosis^51^. Activation of Wnt signaling and Akt maintains the mitochondrial membrane and regulates apoptosis repressor Bcl-xL^52^. Overexpression of IGF-2a and loss of Squint may impair mitophagy and apoptosis in zebrafish embryos. However, IGF-1 induces mitophagy through BNIP3 accumulation in mitochondria, stimulates mitochondrial biogenesis^53^, and protects mitochondria from apoptosis^54^. IGF-IIR, the receptor of IGFII, induces mitophagy through Rab9-dependent alternative autophagy^55^. In contrast, it has been reported that IGF-IIRα disrupts mitochondrial membrane potential, induces perturbation of mitophagy, and can lead to mitochondrial oxidative stress^56^. Further study is needed to clarify these controversial results. *Drfundc1* knockdown impaired mitophagy and apoptosis and resulted in a similar phenotype and midline bifurcation in our experiment, suggesting that midline bifurcation results from impairment of mitophagy and apoptosis during embryogenesis.

In addition to midline bifurcation, *Drfundc1* knockdown led to other severe defects in the anterior section of zebrafish embryos. Knockdown of *Drfundc1* decreased expression of *cyclinD1*, *pax2a*, *opl*, and *neuroD1*, which are involved in development of the neural system^34^–^39^. However, the ways in which these genes are affected remains unclear.

Overall, we found that Fundc1 is critical for proper body axis formation in fish. Fundc1 knockdown induces insufficient mitophagy or autophagy, interferes with apoptosis, and leads to body axis malformation. Interference with Fundc1 expression impacts on genes which are important for typical embryogenesis.

## Materials and Methods

### Ethics

This study was conducted in strict accordance with recommendations for the Regulation for the Management of Laboratory Animals from the Ministry of Science and Technology in China. The animal protocol was approved by the Animal Care and Use Committee of Hubei Province in China (No. SYXK(E)2015-0012).

### Experimental animals

Wild-type zebrafish were kept at 28 °C in circulating water on a 14 h:10 h light:dark cycle and fed twice daily. Fertilized eggs were incubated at 28.5 °C in an embryo medium and staged according to hours post fertilization (hpf) and days post fertilization (dpf), following standard criteria^57^.

### Cell culture and transfection

An ovary cell line (GCO) of grass carp (*Ctenopharyngodon idellus*) was obtained from the National Key Laboratory of Fresh Water Ecology and Biotechnology at the Institute of Hydrobiology, Chinese Academy of Sciences. GCO cells were cultured in M199 medium (Thermo Fisher Scientific, Carlsbad, CA, USA; C11150500BT) and supplemented with 10% inactivated fetal bovine serum (FBS) (Thermo Fisher Scientific)^58^. HEK 293 T cells were cultured in high-glucose Dulbecco’s modified Eagle medium (Thermo Fisher Scientific; C11995500BT) and supplemented with 10% FBS^59^. GCO and 239 T cells were incubated at 28 °C and 37 °C respectively in a humidified atmosphere of 95% air and 5% CO_2_.

A fusion gene of *Drfundc1* with *Cherry* and *His* tags (*Drfundc1-Cherry-His*) was subcloned into pCS2 + vector as pCS2 + -Drfundc1-Cherry-His. pCS2 + -Cherry was constructed as a control. Plasmids were transfected into GCO or 239 T cells using PolyJet (SignaGen Laboratories, Rockville, MD, USA; SL100688) at a cell density of 70–80% in plates, or at a cell density of 50% on a coverslip, following manufacturer’s instructions. Transfection medium was replaced with a fresh complete medium at 12–18 h post-transfection.

### Extraction of mitochondrial protein and Western blotting

We isolated mitochondrial and cytoplasmic proteins from transgenic GCO and 239 T cells transfected with pCS2 + -Drfundc1-Cherry-His and/or pCS2 + -Cherry, strictly following the mitochondria protein extraction kit manual (Jiancheng Bioengineering Institute, Nanjing, China; G006). DrFundc1, Cherry, FUNDC1, COX IV, LC3B, and β-ACTIN were detected by use of Western blotting. Antibodies used were anti-His (Beyotime Biotechnology, Shanghai, China; AH367), anti-Cherry (ABclonal, Wuhan, China; AE002), anti-FUNDC1 (Bioss Inc., Boston, MA, USA; bs-13227R), anti-COX IV (ABclonal; A10098), anti-LC3B (ABclonal; A7198), and anti-β-ACTIN (Bioss Inc.; bsm-33036M).

### Cell staining and fixation

In order to label mitochondria, GCO and 239 T cells were incubated with pre-warmed (37 °C) MitoTracker Green FM probes (Thermo Fisher Scientific; M7514) at a concentration of 200 nmol/L for 40 min under growth conditions in the dark. Nuclei were stained with Hoechst 33258 (Thermo Fisher Scientific; H3569). After staining, the solution was replaced with a fresh pre-warmed culture medium. Images were taken using an EVOS FL auto fluorescence microscope (Thermo Fisher Scientific).

In order to label lysosomes, CellLight Lysosomes-GFP reagent (Thermo Fisher Scientific; C10596) was added to cells in a complete culture medium, following the manufacturer’s instructions, and was gently mixed. Cells were incubated with the reagent overnight (≥16 h) in the dark. Images were taken with a confocal microscope (Leica, Germany; SP5) following cell fixation.

### TUNEL assay

A one-step TUNEL apoptosis assay kit (Beyotime Biotechnology; C1088) was utilized to detect apoptosis in 293 T cells. The TUNEL reaction was carried out according to the protocol^60^ and the manufacturer’s instructions. After fixation with freshly prepared 4% paraformaldehyde (PFA) for 30 min, cells were washed once with phosphate-buffered saline (PBS) and then incubated at room temperature with PBS containing 0.3% Triton X-100 for 5 min. Next, cells were incubated with 100 μL TUNEL detection solution in a 24-well plate at 37 °C for 60 min in the dark. Following Triton X-100 treatment and PBS immersion, positive controls were treated with DNase I reaction solution (Beyotime Biotechnology; C1082) at room temperature for 10 min, while negative controls were incubated without the terminal transferase (TdT) enzyme reaction solution during the labeling reaction. Coverslips were washed three times with PBS and mounted onto slides using antifade mounting medium (Beyotime Biotechnology; P0126). Images were taken with an EVOS FL auto fluorescence microscope (Thermo Fisher Scientific).

### Cell death and cell proliferation assay

An MTT proliferation assay kit (Beyotime Biotechnology; C0009) was used to measure cell viability. Briefly, 293 T cells were seeded into 96-well plates and cultured. Cell viability was measured according to the manual of the kit post-transfection.

Trypan Blue (Thermo Fisher Scientific; T10282) was used to detect dead cells; 10 μl 0.4% Trypan Blue solution was added into 100 μl cell suspensions. Blue staining cells and total cells were counted using a hemocytometer. Cells were also stained with Trypan Blue in plates after fixation with 4% PFA followed by a wash with PBS.

Cell proliferation was also detected with BrdU incorporation^61^. The 293 T cells were seeded onto cover slips and transfected with plasmids, as described above. After transfection, cells were incubated with 10 μmol/L 5-bromo-2′-deoxyuridine (BrdU) (Thermo Fisher Scientific; B23151) in culture medium for 9 h in dark and then fixed with 4% PFA. Cells were washed with a Tris-buffered saline Tween-20 (TBST) containing 0.1% Triton X-100 (TBSTx) after fixing and were incubated in 1.5 mol/L HCl for 30 min to expose antigens. After washing, 5% BSA in TBSTx was applied onto cells for blocking. Cells were then incubated with BrdU monoclonal antibody (ABclonal; A1482) for 12 h at 4 °C. After washing three times with TBSTx, Alexa Flour 488-conjugated AffiniPure goat anti-mouse IgG (H + L) (ABclonal; AS076) was applied for 1 h in the dark. Cells were stained with Hoechst 33258 after washing and mounted onto slides with antifade mounting medium (Beyotime Biotechnology; P0126). Images were taken with an EVOS FL auto fluorescence microscope (Thermo Fisher Scientific).

### Quantitative real-time PCR (qRT-PCR)

Total RNA of different cells, tissues and/or embryos at different stages was extracted with TRIzol reagent (Thermo Fisher Scientific; 15596026) and then reverse-transcribed into complementary DNA (cDNA) with a FastQuant RT Kit (with gDNase; TIANGEN Biotech, Beijing, China; KR106) after digestion of genomic DNA with RNase-free DNase I, following the manufacturer’s instructions.

QRT-PCR was carried out in a reaction volume of 20 μL containing template cDNA, primers, RNase-free H_2_O, and 10 μL of 2х SuperReal premix plus (SYBR Green) (TIANGEN Biotech; FP205) using the following cycle settings: 95 °C for 3 min, followed by 40 cycles of 95 °C for 10 s and 62 °C for 30 s. Samples were analyzed in triplicate, and gene expression values calculated on technical triplicates and biological replicates. Expression levels of target genes were measured and normalized with that of *β-actin* according to the 2-DCt or 2-DDCt calculation method^62^.

Table S1 shows primers used.

### *In situ* hybridization

Whole mount *in situ* hybridization (WISH) was carried out on zebrafish embryos following the protocol reported previously^63^. Sense and antisense digoxigenin (DIG)-labeled RNA riboprobes were produced, as reported previously^25^. Embryos older than 20 hpf were digested with proteinase K and then hybridized with appropriate riboprobes at 70 °C for 12–16 h. After thoroughly washing and blocking, embryos were incubated with anti-DIG antibodies which conjugated with alkaline phosphatase. Embryos were then stained using nitroblue tetrazolium/5-bromo-4-chloro-3-indolyl phosphate (NBT/BCIP). Images were taken under an MZ16F stereomicroscope (Leica) which was equipped with a digital camera. Grey scales of the genes in WISH were quantified depending on embryos’ grey strengths, using the software ImageJ (https://imagej.nih.gov/ij/). Optical density of the areas stained (S) and neighboring background (B) were measured. Signal strengths were calculated as S-B^64^. The mean strength of each gene was obtained from 10 to 15 embryos in each group.

### Knockdown of *Drfundc1*

In this study, shRNAs which interfered with *Drfundc1* were designed online (http://rnaidesigner.thermofisher.com/rnaiexpress/design.do). Two shRNAs – shRNA1 and shRNA2 – targeted nucleotides of *Drfundc1* ORF from 77 to 99 and 217 to 237 respectively. A mismatched shRNA (shRNAmis) as well as a random shRNA (shRNAran) without any target were designed as controls. Vector pSuper-puro (OligoEngine) was used to construct shRNA expression plasmids. shRNAs or pSuper-puro were microinjected into zygotes at a dosage of 200 pg. *Drfundc1* mRNA was synthesized from pCS2 + -Drfundc1 *in vitro* using an mMESSAGE mMACHINE SP6 transcription kit (Thermo Fisher Scientific; AM1340). To repair defects caused by *Drfundc1* shRNA, 400 pg *of Drfundc1* mRNA was microinjected with 200 pg of the shRNA plasmid into zygotes. Embryos with/without injection of pSuper-puro, shRNAmis, and shRNAran were treated as controls.

### Statistical analyses

Data analyses were carried out using SPSS 17.0 (IBM, Armonk, NY, USA). Differences between groups were analyzed using a one-way analysis of variance (ANOVA). A post-hoc Duncan’s multiple range test was utilized to determine significant differences. All data were obtained from at least three independent experiments (*n* ≥ 3) and were described as mean ± standard error (SEM). *P* < 0.05 was considered statistically significant.

## Supplementary information

Supplementary materials
