# Supplementary material for: Fundc1 is necessary for proper body axis formation during embryogenesis in zebrafish
Source: Sci Rep. 2019 Dec 11;9:18910. doi: 10.1038/s41598-019-55415-0 (PMC6906497; doi:10.1038/s41598-019-55415-0)

Fundc1 is necessary for proper body axis formation during embryogenesis in zebrafish

Gongyu Xu, Hao Shen, Emile Nibona, Kongyue Wu, Xiaomei Ke, Md. Abdullah Al Hafiz, Xiaoting Liang, Xueping Zhong, Qingchun Zhou, Chao Qi, Haobin Zhao*

*Hubei Key Laboratory of Genetic Regulation and Integrative Biology, School of Life Sciences,*

*Central China Normal University, Wuhan 430079, Hubei, China*

Table S1. Primers used for the experiments.

| Species | Gene  (GenBank ID) | Primers | Sequence (5′–3′) | Application | |
| --- | --- | --- | --- | --- | --- |
| *Danio rerio* | *fundc1*  (NM_001002711) | fundc1-ORF-F | GGATCCATGGCGGATCGCGGCGAAGAT | Cloning |  |
|  |  | fundc1-ORF-R | CTCGAGTTAGGATGCCAGGCCTAGCAG |  |  |
|  |  | fundc1-RT-F | ATGGCGGATCGCGGCGAAGAT | RT-PCR |  |
|  |  | fundc1-RT-R | TTAGGATGCCAGGCCTAGCAG |  |  |
|  |  | fundc1-qRT-F | ATAGTGGCTATGTGCAGGTGGACT | QRT-PCR |  |
|  |  | fundc1-qRT-R | GGTATTGAGCTCAGGAGCAGCT |  |  |
|  |  | fundc1-probeF | ATGGCGGATCGCGGCGAAGAT | Probe |  |
|  |  | fundc1-probeR | TTAGGATGCCAGGCCTAGCAG |  |  |
|  |  | fundc1-shRNAF1 | GATCCCCGGCATCAATGGTGGAGTCGAGTATTCAAGAGATACTCGACTCCACCATTGATGCCTTTTTA | ShRNA1 |  |
|  |  | fundc1-shRNAR1 | AGCTTAAAAAGGCATCAATGGTGGAGTCGAGTATCTCTTGAATACTCGACTCCACCATTGATGCCGGG |  |  |
|  |  | fundc1-shRNAF2 | GATCCCCGCTACTGCTGTTGGAGGAGGATTC  AAGAGATCCTCCTCCAACAGCAGTAGCTTTTTA | ShRNA2 |  |
|  |  | fundc1-shRNAR2 | AGCTTAAAAAGCTACTGCTGTTGGAGGAGGATCTCTTGAATCCTCCTCCAACAGCAGTAGCGGG |  |  |
|  |  | fundc1-shRNAFmis | GATCCCCATAGAAGATTCTACAGAGTTTGTTTCAAGAGAACAAACTCTGTAGAATCTTCTATTTTTTA | ShRNAmis |  |
|  |  | fundc1-shRNARmis | AGCTTAAAAAATAGAAGATTCTACAGAGTTTGTTCTCTTGAAACAAACTCTGTAGAATCTTCTATGGG |  |  |
|  |  | shRNAFran | GATCCCCTATGCATCGAGCTATGCATCGAGTTCAAGAGACTCGATGCATAGCTCGATGCATATTTTTA | ShRNAran |  |
|  |  | shRNARran | AGCTTAAAAATATGCATCGAGCTATGCATCGAGTCTCTTGAACTCGATGCATAGCTCGATGCATAGGG |  |  |
|  | *β-actin*  (NM_131031) | actin-RT-F | TCCGGTATGTGCAAAGCCGG | RT-PCR |  |
|  |  | actin-RT-R | CCACATCTGCTGGAAGGTGG |  |  |
|  |  | actin-qRT-F | TACCACTGGTATCGTGCTGGACT | QRT-PCR |  |
|  |  | actin-qRT-R | GGTAAGATCACGACCAGCCAGA |  |  |
|  | *ambra1a*  (NM_001281992) | ambra1a-qRT-F | ATGTATCCTGTCCAGTCGAGAGCG | QRT-PCR |  |
|  |  | ambra1a-qRT-R | TGTGTGGAAGCCATTAAAGACCTGTCC |  |  |
|  | *ambra1b*  (NM_001302220) | ambra1b-qRT-F | TGGCAGAGCCAGAAAGTAGAG | QRT-PCR |  |
|  |  | ambra1b-qRT-R | TGGCCCACTAAAGAGTGCAG |  |  |
|  | *atg5*  (NM_001009914) | atg5-qRT-F | TGGTGTGTTGTTCGACCTCC | QRT-PCR |  |
|  |  | atg5-qRT-R | TTTGAGTGCATCGGCCTCTT |  |  |
|  | *atg7*  (XM_021479676) | atg7-qRT-F | GCATTTGATGCGGATGGACC | QRT-PCR |  |
|  |  | atg7-qRT-R | CGCTCCAGATCTCATTGGCT |  |  |
|  | *atg12*  (NM_001246200) | atg12-qRT-F | AGGACGATACAGTCACTCGC | QRT-PCR |  |
|  |  | atg12-qRT-R | TCCGAAACACTCAAAAAGCAC |  |  |
|  | *baxa*  (NM_131562) | baxa-qRT-F | CAGCCGACTCAAGACGTCTTCAT | QRT-PCR |  |
|  |  | baxa-qRT-R | ACCCTGGTTGAAATAGCCTTG |  |  |
|  | *baxb*  (NM_001013296) | baxb-qRT-F | TCGGTGACAAACTCGACCAG | QRT-PCR |  |
|  |  | baxb-qRT-R | CGACCATCTTGGCTGACAGT |  |  |
|  | *bcl2a*  (NM_001030253) | bcl2a-qRT-F | GATGCCTTCGTGGAGATGTACGGT | QRT-PCR |  |
|  |  | bcl2a-qRT-R | CGATGGTCACTCCTGCCAAG |  |  |
|  | *bcl2b*  (XM_001341178) | bcl2b-qRT-F | GCGCTTCAACGCAGTCATAG | QRT-PCR |  |
|  |  | bcl2b-qRT-R | TGCCATCCAGACAGCAATGT |  |  |
|  | *beclin1*  (NM_200872) | beclin1-qRT-F | CGCAGACTGAAAGTGACAAGC | QRT-PCR |  |
|  |  | beclin1-qRT-R | TCTGGCACTCGTTCTCAGTG |  |  |
|  | *caspase3a*  (NM_131877) | caspase3a-qRT-F | CTTTGATCGCAGGACAGGCAT | QRT-PCR |  |
|  |  | caspase3a-qRT-R | AATCTGCGCAACTGTCTGGT |  |  |
|  | *caspase9*  (NM_001007404) | caspase9-qRT-F | CAGATTCTTCAGCGGCACAGGT | QRT-PCR |  |
|  |  | caspase9-qRT-R | ACGTCTGGTTGCCTTGCTCTGT |  |  |
|  | *col2a1a*  (NM_131292) | col2a1a-probe-F | CACAGGTCAATGGGCCAAGACTGT | Probe |  |
|  |  | col2a1a-probe-R | CATTGGTAGTGCTCGCATGTTCGGT |  |  |
|  | *col8a1a*  (NM_001142374) | col8a1a-probe-F | CCTTGATGGTGTGAAAGCTGGCTAC | Probe |  |
|  |  | col8a1a-probe-R | GAGGAGTGGACAAATTGTCCTGCAT |  |  |
|  | *cyclinD1*  (NM_131025) | cyclinD1-qRT-F | TGCCTATACATCAGAGCTCCAAGCAG | QRT-PCR |  |
|  |  | cyclinD1-qRT-R | GCAACACTGCCTGCTGCAATCATG |  |  |
|  |  | cyclinD1-probe-F | GTGCATATACACGGACAACTCTGTCCG | Probe |  |
|  |  | cyclinD1-probe-R | GAGATGTGCTGCTGAGCTTGTCTGAG |  |  |
|  | *fox3a*  (NM_001009988) | fox3a-qRT-F | AGCCTGCCAGTGTCACAGAAGG | QRT-PCR |  |
|  |  | fox3a-qRT-R | TGGAGTCGCTGCATGCTGCTAG |  |  |
|  | *neuroD1*  (NM_130978) | neuroD1-qRT-F | GTCGCAGGATGCCTCCAACTGAAC | QRT-PCR |  |
|  |  | neuroD1-qRT-R | GTGAGAGCTGTCCATTGTACCGTACG |  |  |
|  |  | neuroD1-probe-F | AGCTCTCCAAGATCGAGACGCTCC | Probe |  |
|  |  | neuroD1-probe-R | GTGAGAGCTGTCCATTGTACCGTACG |  |  |
|  | *opl*  (NM_130933) | opl-qRT-F | AGCACTACGCTTCGACTCAGTTGC | QRT-PCR |  |
|  |  | opl-qRT R | TGCTCTGGTTCGATCCATTTGCAG |  |  |
|  |  | opl-probe-F | ATGGATCGAACCAGAGCAGCTGACG | Probe |  |
|  |  | opl-probe-R | GTCAGAGGTATGGACGTGCATGTG |  |  |
|  | *p53*  (NM_001271820) | p53-qRT-F | GTGCTTGAAGAACAGCCTCAGCCAT | QRT-PCR |  |
|  |  | p53-qRT-R | TCAGGTCCGGTGAATAAGTGCAAGT |  |  |
|  | *p62*  (NM_001312913) | p62-qRT-F | TGGGTTTGGCTCTTGTGAAGGATGAC | QRT-PCR |  |
|  |  | p62-qRT-F | GTGAGGTGTAGTGAACGGAAACCCAG |  |  |
|  | *pax2a*  (NM_131184) | pax2a-qRT-F | CACCTCTACACTTGCTGGAATGGTC | QRT-PCR |  |
|  |  | pax2a-qRT-R | GTCATAGGCAGTGGCAGCAGTG |  |  |
|  |  | pax2a-probe-F | GTCTGTCAACCAGCGTCAGCTCAG | Probe |  |
|  |  | pax2a-probe-R | GTCATAGGCAGTGGCAGCAGTGG |  |  |
|  | *Shha*  (NM_131063) | shha-probe-F | TCCTGTATCAAATGGGAACGTGGCT | Probe |  |
|  |  | shha-probe-R | CAGAATGTCACAACCTGTCATGAAGGT |  |  |
| *Homo sapiens* | *β-ACTIN*  (NM_001101) | ACTIN-qRT-F | CCACGAAACTACCTTCAACTCC | QRT-PCR |  |
|  |  | ACTIN-qRT-R | CTCAGGAGGAGCAATGATCTTG |  |  |
|  | *ATG5*  (NM_001286106) | ATG5-qRT-F | AGGCACACCACTGAAATGGCATTATCC | QRT-PCR |  |
|  |  | ATG5-qRT-R | CCTTAGATGGACAGTGCAGAAGGTC |  |  |
|  | *ATG7*  (NM_001349232) | ATG7-qRT-F | AGCTGAACGAGTATCGGCTGGATG | QRT-PCR |  |
|  |  | ATG7-qRT-R | GTGTTCCAATAGCTGGGCAGCAAC |  |  |
|  | *ATG12*  (NM_004707) | ATG12-qRT-F | GCTAAAGGCTGTGGGAGACACTC | QRT-PCR |  |
|  |  | ATG12-qRT-R | GAAGTCAATGAGTCCTTGGATGGTTCG |  |  |
|  | *BAX*  (NM_001291428) | BAX-qRT-F | CAGGGTTTCATCCAGGATCGAGC | QRT-PCR |  |
|  |  | BAX-qRT-R | TCCTCTGCAGCTCCATGTTACTGTC |  |  |
|  | *BCL2*  (NM_000657) | BCL2-qRT-F | GAGTTCGGTGGGGTCATGTGTGT | QRT-PCR |  |
|  |  | BCL2-qRT-R | ATCCTGGATCCAGGTGTGCAG |  |  |
|  | *BECLIN1*  (NM_003766) | BECLIN1-qRT-F | CCATGGAGAACCTCAGCCGAAG | QRT-PCR |  |
|  |  | BECLIN1-qRT-R | TGAGCTGAGTGTCCAGCTGGTC |  |  |
|  | *CASPASE3*  (NM_004346) | CASPASE3-qRT-F | TCGGTCTGGTACAGATGTCGATGC | QRT-PCR |  |
|  |  | CASPASE3-qRT-R | CCTTCTTCACCATGGCTCAGAAGC |  |  |
|  | *LC3B*  (NM_02281) | LC3B-qRT-F | ATGCCGTCGGAGAAGACCTTCAAGC | QRT-PCR |  |
|  |  | LC3B-qRT-R | ACAGGAAGCTGCTTCTCACCCTTG |  |  |

Fundc1 is necessary for proper body axis formation during embryogenesis in zebrafish

Gongyu Xu, Hao Shen, Emile Nibona, Kongyue Wu, Xiaomei Ke, Md. Abdullah Al Hafiz, Xiaoting Liang, Xueping Zhong, Qingchun Zhou, Chao Qi, Haobin Zhao*

*Hubei Key Laboratory of Genetic Regulation and Integrative Biology, School of Life Sciences,*

*Central China Normal University, Wuhan 430079, Hubei, China*


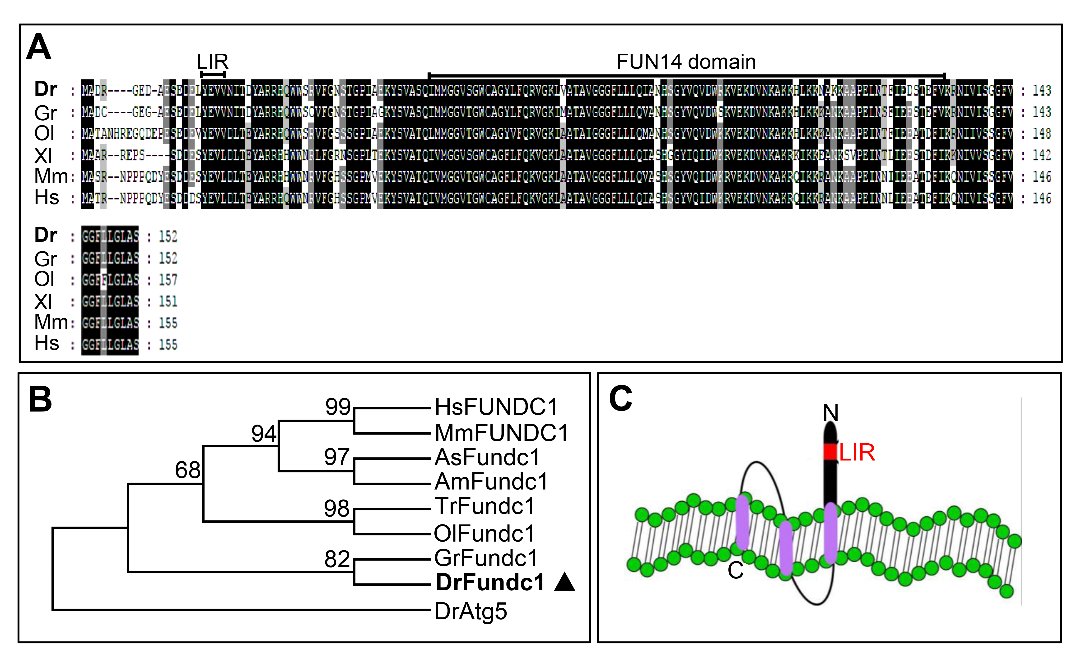
Fig S1. Sequence analyses of DrFundc1. (A) Multiple alignment of selected Fundc1 proteins. Conserved amino acids are highlighted in black. Conserved LIR motif and FUN14 domain are shown above sequences. (B) Phylogenetic tree of Fundc1 proteins established using MEGA6 via maximum likelihood method. Zebrafish Atg5 is used as outgroup. (C) Schematic representation of DrFundc1 in mitochondria as a transmembrane protein with the LIR motif, YEVV, in the N-terminal (N). C, C-terminal.

Fig. S2. *In vitro* study of DrFundc1 in GCO cells by gene transfer with the plasmid pCS2+-Drfundc1-Cherry-His. (A) Co-localization of DrFundc1 (red) with mitochondria. Mitochondria (green) were labeled by MitoTracker Green (Mito). Nuclei (blue) were stained with Hoechst 33258. Scale bar, 100 μm. (B) Use of Western blot for the mitochondrial protein shows DrFundc1 in mitochondria by anti-His antibody. Total mitochondrial protein was extracted from transgenic cells. Sizes (kD) of the marker are labeled on the left. The clear band shows a fusion protein of DrFundc1-Cherry-His at approximately 44 kD. (C) The arrows point to particles related to colocalization of DrFundc1 (red) and lysosomes (green). Lysosomes were stained by CellLight Lysosomes-GFP (Lyso). Inserts are the counterparts in high magnification. Scale bar, 20 μm. (D) Forced expression of Drfundc1 caused cell death by transgene of pCS2+-Drfundc1-Cherry-His, while pCS2+-Cherry was transferred as control. Dosages were 0, 300, 400 and 500 ng for each well of a 24-well plate. Images were taken in bright and dark fields. Scale bar, 200 μm. (E) Cell numbers were calculated 48 h after gene transfer. The horizontal axis indicates number of plasmids. Asterisks show significances between cells with Drfundc1 and Cherry.


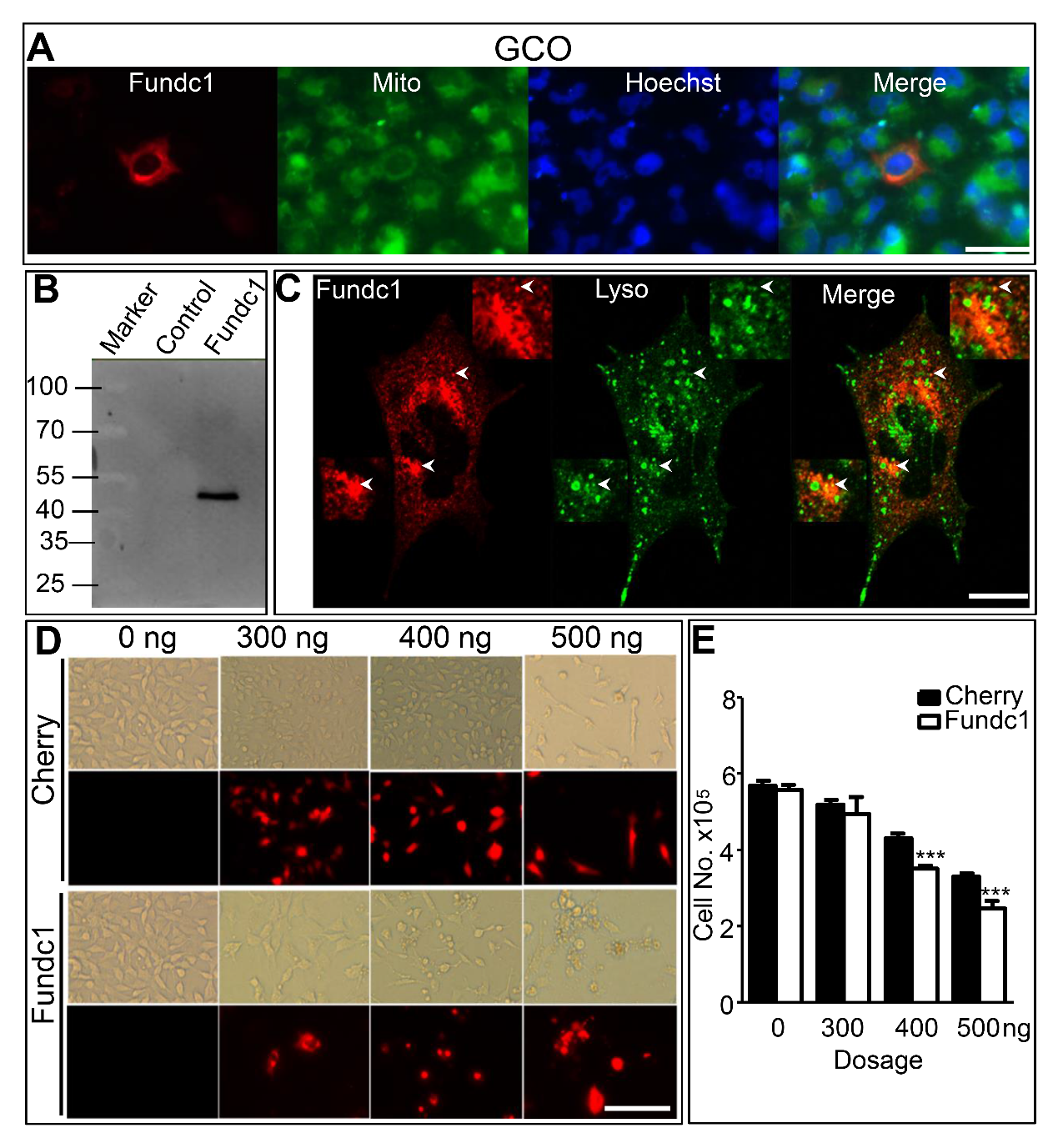


Fig. S3. DrFundc1 damaged cell growth and cell proliferation in transgenic 293T cells. (A) DrFundc1 caused cell death. The 293T cells were transfected with 500 ng of pCS2+-Drfundc1-Cherry-His or pCS2+-Cherry plasmids in a 24-well plate. Forced expression of DrFundc1 caused morphological change and cell floating, while Cherry had no effect. Scale bar, 200 μm. (B) DrFundc1 decreased the numbers of 293T cells in 48 h. (C) Cell proliferation was detected using BrdU incorporation. BrdU antibody (green) was used to detect BrdU in cell nuclei after fixation. Nuclei (blue) were stained with Hoechst 33258.


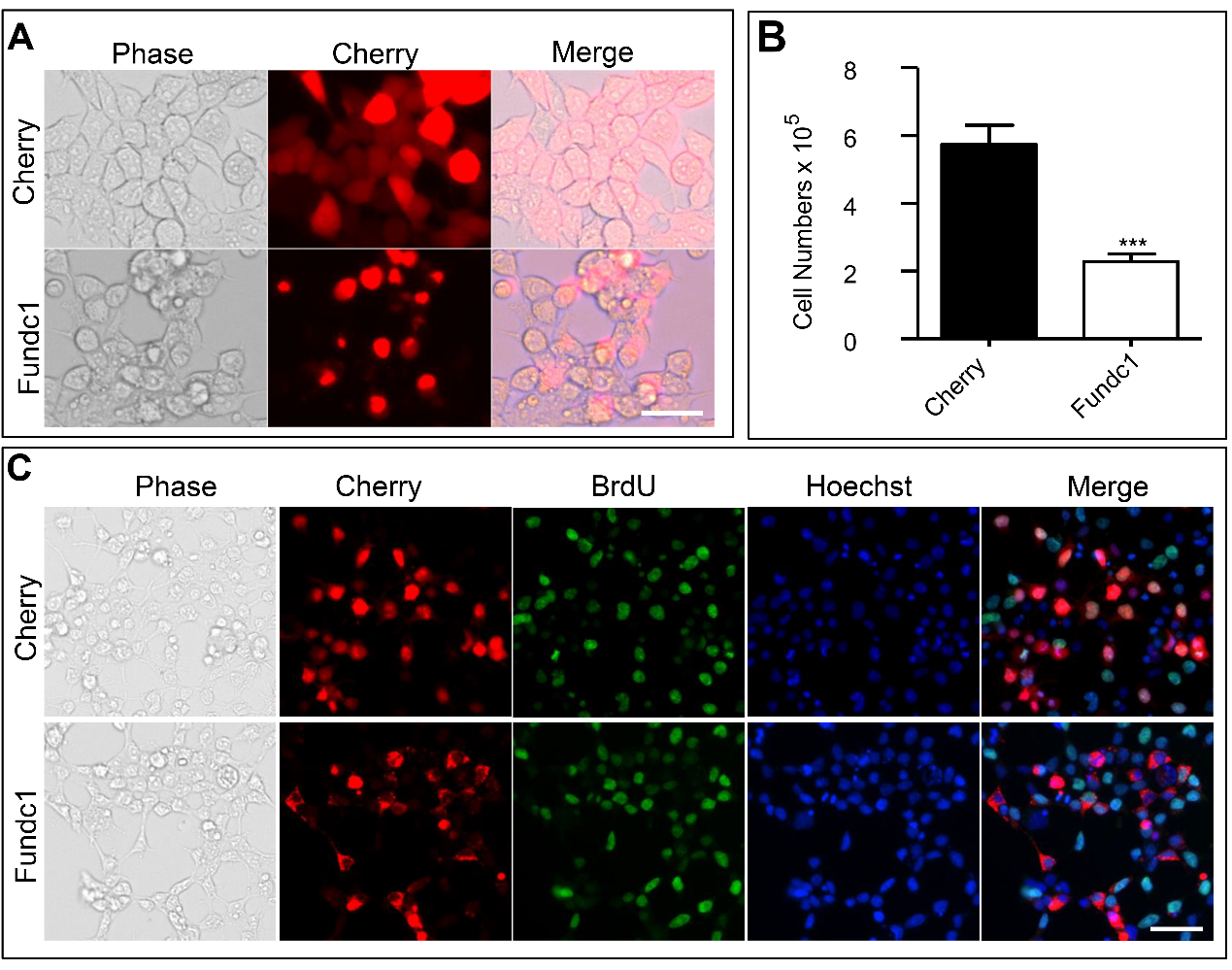


Fig S4. Expression of *Drfundc1* in adult tissues (A) and embryos (B) normalized with *β-actin*. (C) WISH of *Drfundc1* in zebrafish embryos. Embryos at 1-cell, 4-cell, 8-cell, gastrula (6 hpf), 24 hpf, 48 hpf, and 72 hpf, are oriented with side view. hpf, hours post fertilization.


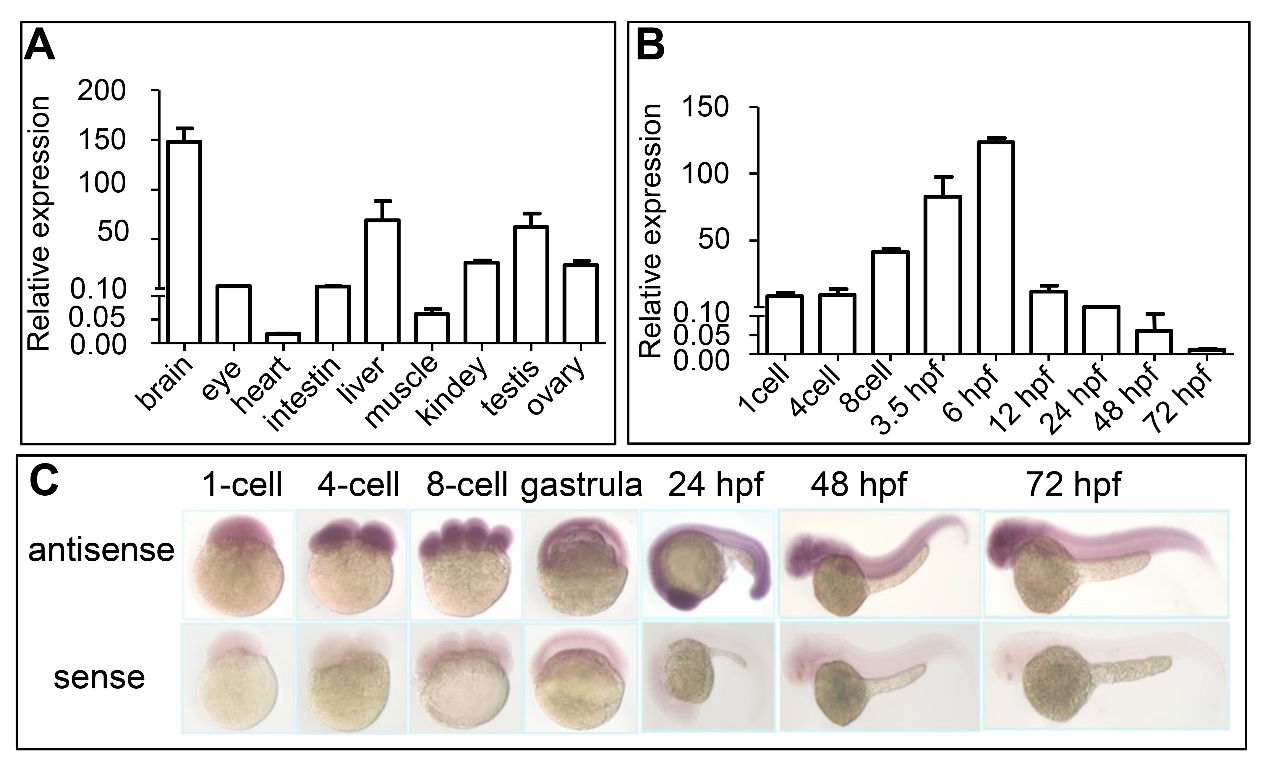


Fundc1 is necessary for proper body axis formation during embryogenesis in zebrafish

Gongyu Xu, Hao Shen, Emile Nibona, Kongyue Wu, Xiaomei Ke, Md. Abdullah Al Hafiz, Xiaoting Liang, Xueping Zhong, Qingchun Zhou, Chao Qi, Haobin Zhao*

*Hubei Key Laboratory of Genetic Regulation and Integrative Biology, School of Life Sciences,*

*Central China Normal University, Wuhan 430079, Hubei, China*

The original image of Fig. 1A Part1. Western blotting with anti-Cherry to detect Cherry and

DrFundc1-Cherry, and anti-LC3B antibody to detect LC3B-I and LC3B-II. Mitochondrial proteins (Mitochondria) and cytoplasmic proteins (Cytoplasm) were extracted from transgenic cells with pCS2+-Drfundc1-Cherry-His or pCS2+-Cherry. Marker sizes are labeled at the left beside the image. Blue arrows, DrFundc1-Cherry (~ 45 kD); white arrows, Cherry (~ 28 kD, ~50 kD); red arrow, LC3B-I (~ 16 kD); black arrow, LC3B-II (~ 14 kD). Other unmarked bands are non-specific bands due to the specificities of the antibodies. The bands of Cherry between 40 to 55 kD may be the dimer of Cherry (Wu, Chen, & Muller, 2009).

Mitochondria

# Cytoplasm

Mitochondria

# Cytoplasm

- + - +

+ - + -

- + - +

*Drfundc1-cherry*

*cherry*

+ - + -


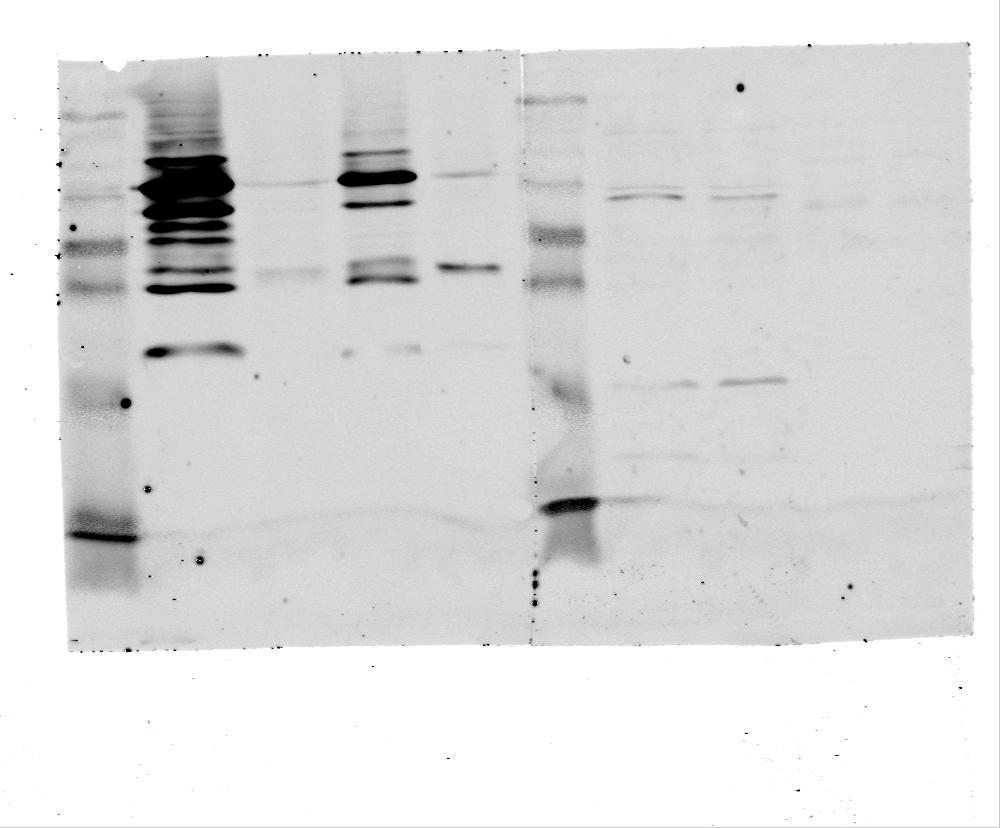


Drfundc1

cherry

Drfundc1

cherry

Mito

Cytosol

100

55

40

35

25

15

10

**Reference**

Wu, B., Chen, Y. & Muller, J. D. Fluorescence Fluctuation Spectroscopy of mCherry in Living Cells. Biophys J. **96**, 2391-2404 (2009).

The original image of Fig. 1A Part 2. Western blotting with anti-FUNDC1 and anti-COX IV to detect endogenous FUNDC1 and COX IV respectively. Marker sizes are labeled at the left. White arrows, FUNDC1 (~ 17 kD); red arrows, COX IV (~ 20 kD). Other unmarked bands are non-specific bands due to the specificities of the antibodies.

Mitochondria

# Cytoplasm

Mitochondria

# Cytoplasm

+ - + -

*Drfundc1-cherry*

- + - +

+ - + -

- + - +

*cherry*


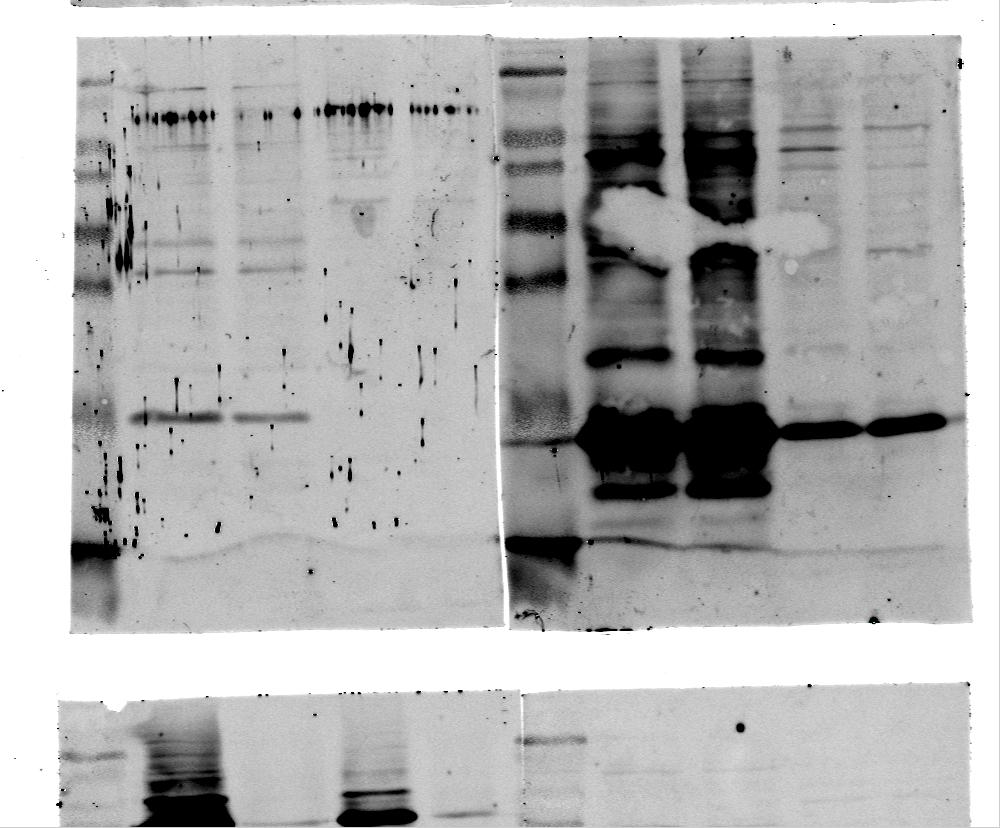


130

40

55

100

35

25

15

10

The original image of Fig. 1A Part 3. Western blotting with anti-ACTIN to detect β-ACTIN (~ 42 kD, red arrow)

Mitochondria

# Cytoplasm

+ - + -

*Drfundc1-cherry*

- + - +

*cherry*


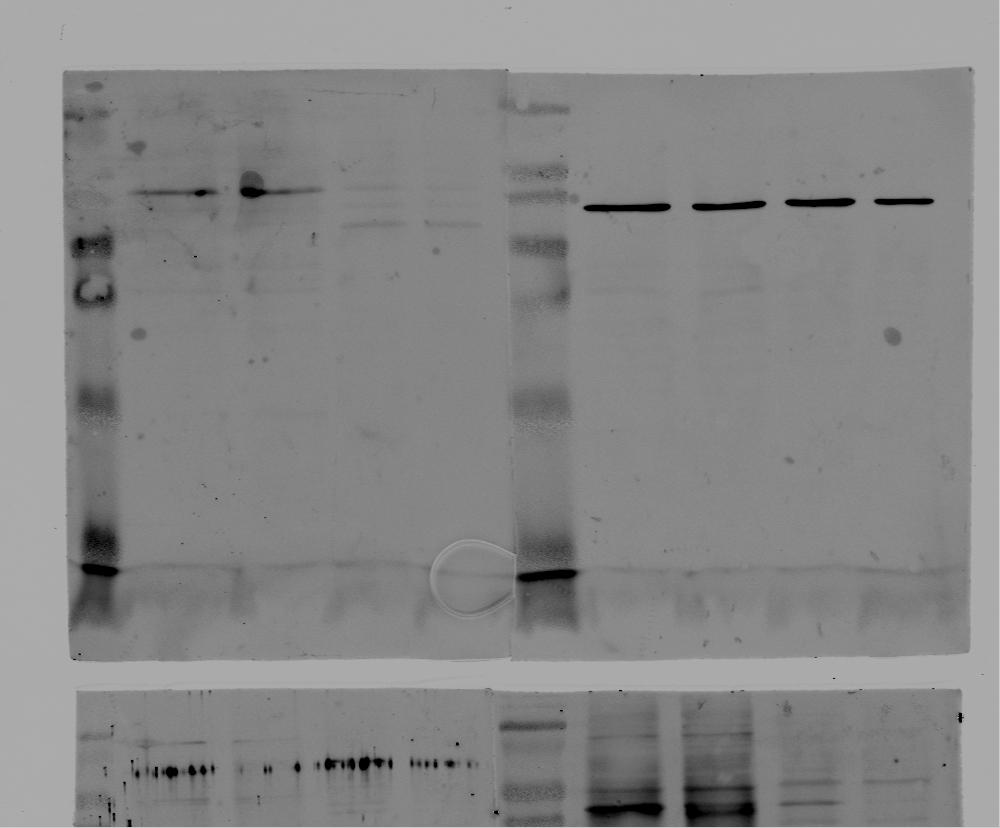


10

25

100

35

15

40

55

The original image of Fig. S2B, Western blotting with anti-His antibody to detect DrFundc1.

Mitochondrial proteins (Mitochondria) and total proteins of the cells (Cell) were extracted from transgenic cells with pCS2+-Drfundc1-Cherry-His. Marker sizes: 100, 70, 55, 40, 35, 25 kD from up to down.

Control

Marker

Empty

Cell

Mitocondria

Control

Mitocondria

Mitocondria

Control

Marker


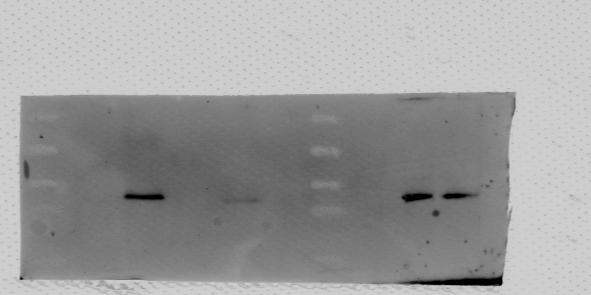

Supplement: Supplementary file 1 — Supplementary materials [file 41598_2019_55415_MOESM1_ESM.docx]
